# Supplementary material for: Ceruloplasmin replacement therapy ameliorates neurological symptoms in a preclinical model of aceruloplasminemia
Source: EMBO Mol Med. 2017 Nov 28;10(1):91–106. doi: 10.15252/emmm.201708361 (PMC5760856; doi:10.15252/emmm.201708361)
Supplement: Supplementary file 1 — Appendix [file EMMM-10-91-s001.docx]

**Appendix**

**Ceruloplasmin replacement therapy ameliorates neurological symptoms in a preclinical model of aceruloplasminemia**

Zanardi A, Conti A, Cremonesi M, D'Adamo P, Gilberti E, Apostoli P, Cannistraci CV, Piperno A, David S, Alessio M.

**Inventory of Appendix data:**

**Appendix Figures S1-S4**

**Appendix Table S1**

**Appendix Figure S1**

Analysis of sialylation status of human purified ceruloplasmin.

Sialylation status analysis of administered human Cp was performed with DIG-Glycan Differentiation Kit (Roche, 11 210 238 001) and neuraminidase treatment. Human purified Cp untreated or treated with 40 mU of *Vibrio cholerae* neuraminidase (Roche Diagnostics) in 50 mM sodium acetate (pH 5.5) in order to remove sialic acids, was resolved by SDS-PAGE and analyzed by Western blot using lectins *Sambucus nigra* agglutinin (SNA) and *Maackia amurensis* agglutinin (MAA), which specifically recognize sialic acid. Membranes were incubated with lectins conjugated with digoxigenin (DIG) (SNA 0.5 μg/mL, MAA 2.5 μg/mL) and reactivity was revealed by incubation with HRP-conjugated rabbit-anti-DIG antibody (Dako, P5104) (working dilution 1:1000) followed by ECL-reaction and films exposure.


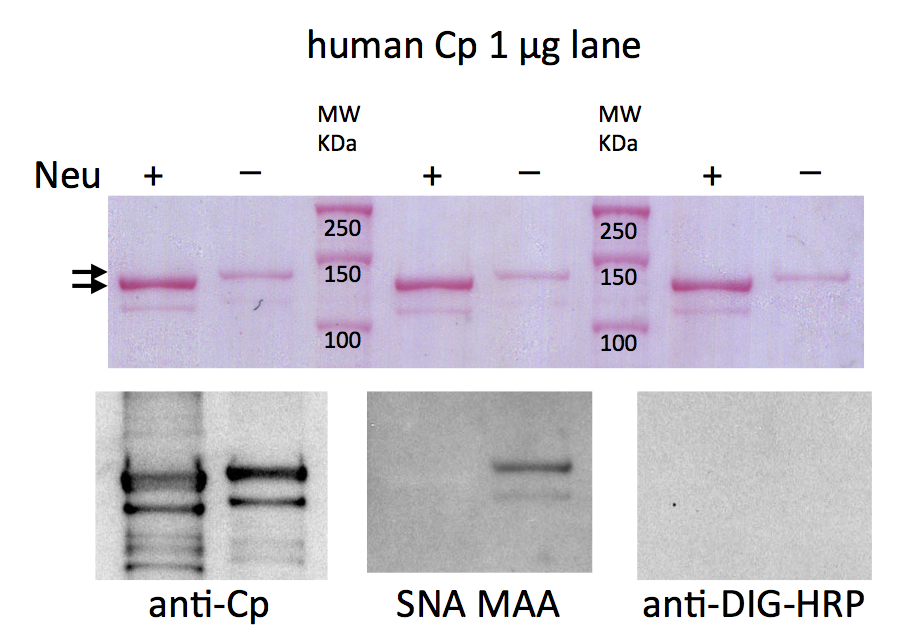


**Figure S1.** Analysis of the human purified Cp sialylation status. The reactivity of *Sambuca Nigra* agglutinin (SNA) and *Maackia amurensis* agglutinin (MAA) lectins specific for sialic acids, tested on human purified Cp (1 μg) untreated or treated with neuraminidase (Neu), showed loss of lectins reactivity, which in turn indicated that administered Cp is sialylated. The upper panel shows the red ponceau staining of the purified Cp transferred to nitrocellulose. A slightly decrease in relative mass is observable upon neuraminidase treatment (indicated by the arrows). The staining with an anti-Cp antibody confirmed the integrity of the protein, while control anti-DIG secondary antibody reactivity was used as control.

**Appendix Figure S2**

Evaluation of protein carbonylation in brain

Oxidation level of the total protein contents in brain was measured using OxyBlot^TM^ Detection kit (Millipore). Protein oxidative modifications lead to the introduction of carbonyl groups into amino acid side chains. The assay is based on the derivatization of carbonyl groups to 2,4-dinitrophenylhydrazone (DNP) by reaction with 2,4-dinitrophenylhydrazine (DNPH). The DNP-derivatized proteins are recognized by a specific anti-DNP antibody in Western blot. Brain homogenates (20 μg of total protein) were derivatized according to manufacturer instruction, proteins were then resolved on 10%-acrylamide SDS-PAGE and transferred onto a nitrocellulose membrane. Western blot was performed using anti-DNP antibody (1:150) and a secondary HRP-conjugated antibody. Signals were detected using ECL™ reagent (GE-Healthcare) followed by films exposure, and densitometric analysis was performed using ImageJ software (Rasband, W.S., ImageJ, U. S. National Institutes of Health, Bethesda, Maryland, USA, http://imagej.nih.gov/ij/, 1997-2016) normalizing the signal to the total protein loaded and to actin expression detected using anti-actin antibody (Sigma).


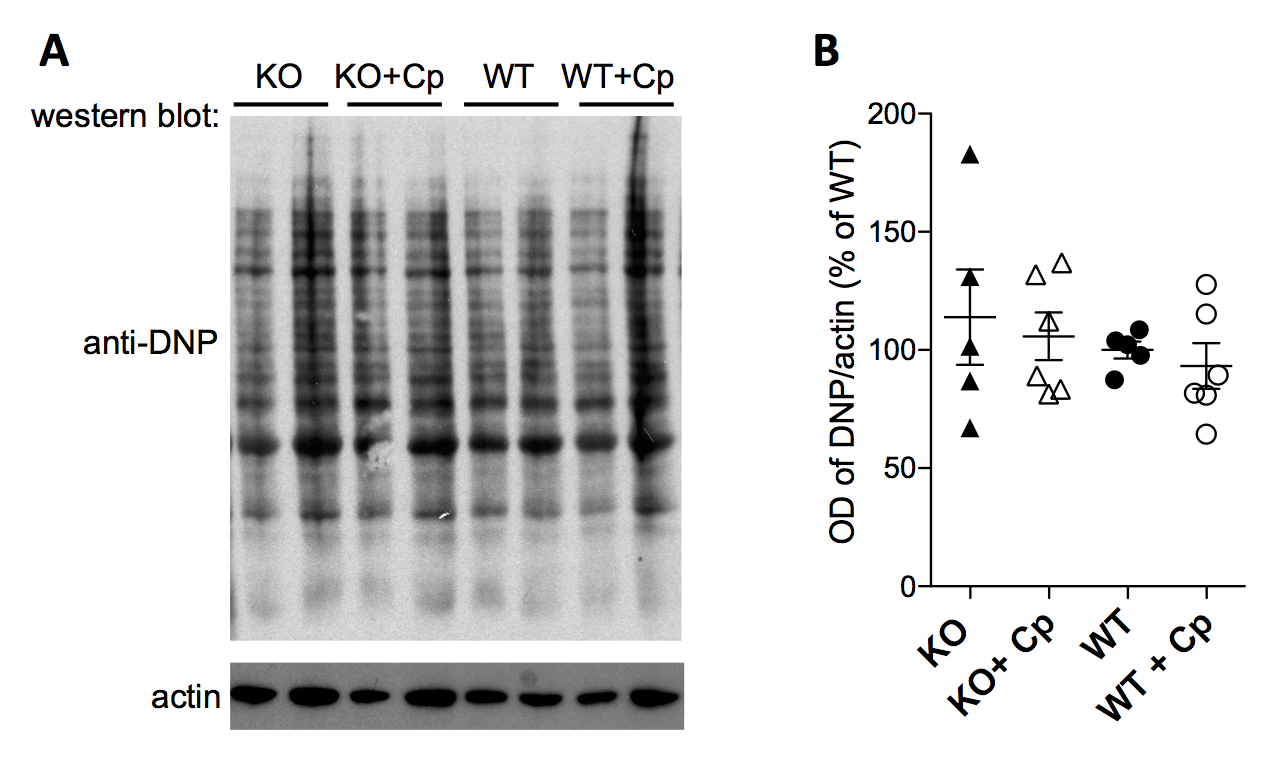


**Figure S2.** Measurements of the total protein carbonylation level in brain (**A**) Representative western blot of DNP detection in brain homogenates; (**B**) Optical density (OD) analysis of DNP signals normalized for actin, did not showed significant differences among groups, indicating that at 10 months of age in CpKO mice is not already present a pro-oxidant environment that might affect Cp structure and activity. Data are presented as mean ± SEM of signal percentage compared to the average value of CpWT mice. Each dot corresponds to one animal.

**Appendix Figure S3**

Analysis of total iron content in liver. Quantitative analysis of total iron content in liver of 10 month old mice was performed by inductively coupled plasma mass spectrometry (ICP-MS) on dry tissue as described in Materials and Methods.

**Figure S3.** CpKO mice showed significant iron accumulation in liver compared to WT mice Cp, and Cp treatment was able to induce a reduction of iron accumulation with a trend that, however, did not reach statistical significance. Data are presented as mean ± SEM and statistical significance was evaluated by Student's t test; each dot corresponds to one animal. These data suggest that the proposed enzyme replacement therapy might be also efficacious on Acp systemic symptoms.

**Appendix Figure S4**

Analysis of brain iron deposition. Modified Perl’s histochemistry was performed on fixed brain sections from CpKO and WT mice as described in Materials and Methods. Images were acquired with Nuance® FX multiplex image system (PerkinElmer) at different wavelength corresponding to the chromophore emission.

**Figure S4.** Iron accumulation occurs in choroid plexus but not in microvasculature endothelial cells of CpKO mice. A brain iron accumulation (brown staining) occurred in the choroid plexus epithelial cells of CpKO mice compared to WT mice (empty arrows and figure 5 in the text). While, no staining for iron was observed in endothelial cells of the brain microvasculature (black arrows). Bottom panels show enlargement of the regions including endothelia microvasculature. Images acquired at different wavelength were computationally reassembled in pseudo color (iron= brown; cells= blue), and their corresponding gray scale images are also shown. Scale bars= 50 μm and 25 μm for low and high magnification, respectively.

**Appendix Table S1**

Measured parameters used for multivariate dimensional reduction analysis
